# In Situ Visualization of the Local Photothermal Effect Produced on α-Cyclodextrin Inclusion Compound Associated with Gold Nanoparticles

**DOI:** 10.1186/s11671-016-1322-z

**Published:** 2016-04-07

**Authors:** Nataly Silva, Camila Muñoz, Jordi Diaz-Marcos, Josep Samitier, Nicolás Yutronic, Marcelo J Kogan, Paul Jara

**Affiliations:** Department of Chemistry, Universidad de Chile, Las Palmeras, 3425 Santiago, Chile; Centros científicos y tecnológicos (CCiTUB) y NanoDivulga, Universidad de Barcelona, Lluís Solé i Sabaris 1-3, Barcelona, 08028 Spain; Nanobioengineering Laboratory, Institute for Bioengineering of Catalonia (IBEC), BaldiriReixac,10-12, Barcelona, 08028 Spain; Centro de Investigación Biomédica en Red de Bioingeniería, Biomateriales y Nanomedicina (CIBER-BBN), Maria de Luna, 11, 50018 Zaragoza, Spain; Department of Electronics, Barcelona University (UB), Martí I Franques, 1, Barcelona, 08028 Spain; Department of Pharmacological and Toxicological Chemistry, Universidad de Chile, Sergio Livingston 1007 Santiago, Chile; Advanced Center for Chronic Diseases (ACCDiS), Santiago, Chile

**Keywords:** Gold nanoparticles, Plasmonic heating, Cyclodextrin inclusion compound, Guest migration

## Abstract

Evidence of guest migration in α-cyclodextrin-octylamine (α-CD-OA) inclusion compound (IC) generated via plasmonic heating of gold nanoparticles (AuNPs) has been studied. In this report, we demonstrate local effects generated by laser-mediated irradiation of a sample of AuNPs covered with inclusion compounds on surface-derivatized glass under liquid conditions by atomic force microscopy (AFM). Functionalized AuNPs on the glass and covered by the ICs were monitored by recording images by AFM during 5 h of irradiation, and images showed that after irradiation, a drastic decrease in the height of the AuNPs occurred. The absorption spectrum of the irradiated sample showed a hypsochromic shift from 542 to 536 nm, evidence suggesting that much of the population of nanoparticles lost all of the parts of the overlay of ICs due to the plasmonic heat generated by the irradiation. Mass spectrometry matrix-assisted laser desorption/ionization-time-of-flight (MALDI-TOF) performed on a sample containing a collection of drops obtained from the surface of the functionalized glass provided evidence that the irradiation lead to disintegration of the ICs and therefore exit of the octylamine molecule (the guest) from the cyclodextrin cavity (the matrix).

**Electronic supplementary material:**

The online version of this article (doi:10.1186/s11671-016-1322-z) contains supplementary material, which is available to authorized users.

**Graphical Abstract:**
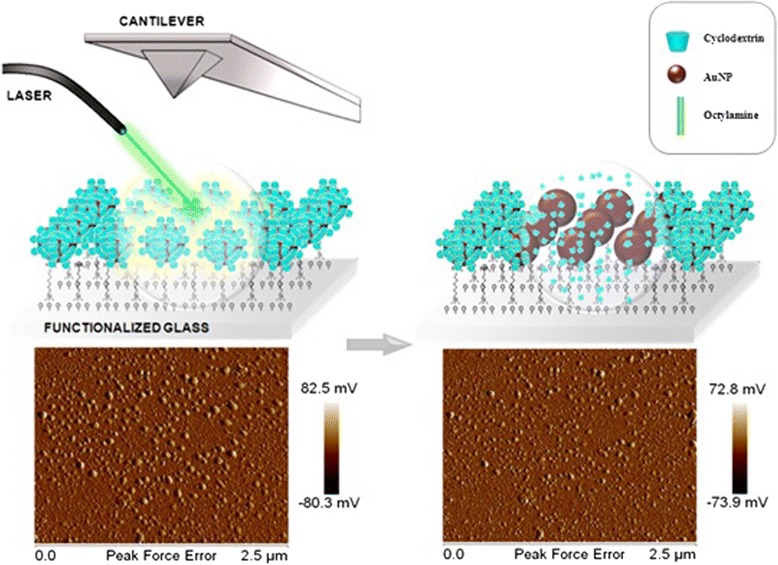
Atomic Force Microscopy observation of the disintegration of a cyclodextrin inclusion compound by gold nanoparticles photothermal effect

## Background

Due to the strong electric fields at the surface, absorption and scattering of electromagnetic radiation by noble metal nanoparticles is enhanced robustly. These unique properties provide the potential for designing novel optically active reagents. The use of nanoparticles in medicine is one of the important directions being taken by current research in nanotechnology. Their applications in drug delivery, cancer cell diagnostics and therapeutics have been the active fields of research [1–6]. Plasmon resonance of gold nanostructures is of great interest for photothermal properties and optical imaging due to their remarkable capacity to absorb and scatter light at visible and near-infrared (near-IR) regions. These optical properties depend on the nanoparticle size, shape, and dielectric environment and enable their application in novel imaging techniques and as sensing probes [1, 2, 7, 8]. Gold nanoparticles (AuNPs) convert optical energy into heat via non-radiative electron relaxation dynamics, which endow them with intense photothermal properties. These localized heating effects can be directed toward the eradication of diseased tissue, providing a non-invasive alternative to surgery. Colloidal gold is well known to be biologically inert and has been used in vivo since the 1950s, particularly as an adjuvant in radiotherapies; however, the use of these nanoparticles as photothermal agents is relatively recent [2]. Additionally, AuNPs are useful for drug delivery by using laser irradiation to control spatial and temporal drug release [9].

On the other hand, in the nanoscience field, cyclodextrins (CDs) have been used to prepare AuNPs capped with thiolated α- and β-CDs [10] for the formation of AuNPs by femtosecond laser ablation [11] and also to prepare AuNPs by chemical reduction in presence of unmodified CDs [12]. The CD-ICs, particularly those leading to supramolecular self-assemblies, continue to be a fascinating topic in modern organic chemistry because they serve as models for understanding molecular recognition [13–16] and as precursors for designing novel nanomaterials [17].

In solid α-CD-ICs with alkylated guests, the functional group of the guest molecule may be located at the extreme boundary of a CD unit in the electron-dense space, and the alkyl chain may be located in the apolar and electron-poor zone of the CD cavities [18–20]. These functional groups (–SH, –COOH, –NH_2_) are available to interact with the particles thus stabilizing them [21].

The migration of alkyl thiol molecules included in the cavities of the channel-type structure of α-CD-IC in the presence of AuNPs by powder X-ray diffraction has been reported [22].

Moreover, the atomic force microscopy (AFM) technique has been used to monitor changes in the different substrates produced by photothermal processes, such as DNA fragmentation [23–25].

**Fig. 1:**
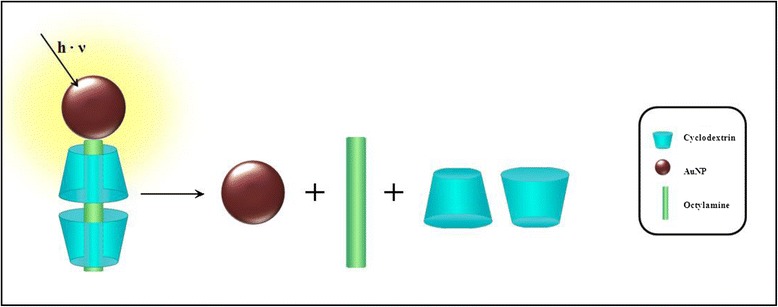
A schematic representation of an IC attached to the surface of an AuNPs. Laser light is absorbed by the AuNPs and transformed into local heat, which dissipates into the environment and induces the release of the guest of the cyclodextrin cavity

In this study, we report for the first time a study of the effects of green-laser irradiation on AuNPs conjugated to a CD supramolecular structure assembled with derivatized slides using an in situ monitoring AFM strategy to demonstrate that the heat generated in the irradiated nanostructure is transferred to the supramolecular structure resulting in the disintegration of the AuNPs-IC system and the exit of the guest molecule from the CD cavity (Fig. 1). This study is relevant for the future applications of these systems in drug delivery.

## Methods

### Synthesis of Colloidal AuNPs

The gold colloid was synthesized based on the Turkevich method with slight modifications [26]. Prior to the synthesis, all glassware was thoroughly cleaned by soaking in aqua regia (comprising 3 parts HCl (Merck) to 1 part HNO_3_ (Merck)) and rinsing with Milli-Q water (18 MΩ Millipore Nanopure purification system).

In a 250-mL round-bottom flask equipped with a condenser, 100 mL of an aqueous HAuCl_4_ solution (1 mM) (HAuCl_4_·3H_2_O; Sigma-Aldrich) was brought to a rolling boil with vigorous stirring. As quickly as possible, 10 mL trisodium citrate dihydrate (Na_3_C_6_H_5_O_7_·2H_2_O; Sigma-Aldrich) solution (38.8 mM) was added to the solution with constant stirring. The solution was heated for an additional 30 min and left at room temperature. The solution was then filtered through a 0.45-μm filter membrane of cellulose acetate thus obtaining 12-nm diameter AuNPs.

### IC Preparation

ICs were obtained using octylamine (OA; Sigma-Aldrich) and a saturated solution of α-cyclodextrin (α-CD; Sigma-Aldrich) in Milli-Q water at room temperature. The amine/cyclodextrin molar ratios used in the experiments were 2:1. The crystals were filtered and dried under vacuum [27, 28].

### Glass Derivatization

Samples were prepared following protocols described previously [29–31]. The glass slides (1 cm × 1 cm) were cleaned for 30 min in a bath “piranha solution” comprising 4 parts sulfuric acid (H_2_SO_4_; Merck) to 1 part hydrogen peroxide (H_2_O_2_; Merck) at 60 °C. After rinsing with methanol (CH_3_OH) spectrophotometric grade (Merck), the slides were dipped for 24 h in vials containing (3-aminopropyl)-trimethoxysilane (APTMS; Sigma-Aldrich) in CH_3_OH solution (1 APTMS:4 CH_3_OH).

### Glass Functionalization

The slides were rinsed with Milli-Q water and immersed in colloidal gold solution at room temperature for 24 h. A final Milli-Q water rinse concluded the derivatization process.

### AuNP IC Conjugation

The functionalized slides with AuNPs were immersed in IC solution in dimethyl sulfoxide (DMSO; Sigma-Aldrich) (prepared from dissolving IC crystals) for 24 h. Finally, the slides were rinsed with Milli-Q water.

### Irradiation of the Functionalized Glass with AuNPs and Conjugation to the IC

A water drop was added to the IC/conjugated glass, and the system was irradiated using a continuous wave laser (532 nm wavelength, potency 45 mW) for 5 h.

### Atomic Force Microscopy (AFM)

Experiments were performed to determine changes in height of the particles during the several stages of glass functionalization. All measurements were performed in situ at room temperature using a MultiMode with electronic NanoScope V (Bruker) with an incorporated fluid cell. The images were obtained in PeakForce Tapping mode, using SNL model probes from Bruker, a spring constant of 0.35 N/m, and nominal tip radius of curvature of 10 nm. The scan-line speed was optimized to between 0.5 and 1 Hz with a pixel number of 512 × 512.

AFM micrographs were recorded every 15 min, over a sample irradiated in situ for a total period of 5 h, the time it took for the drop to vaporize (Additional file 1: Figure S1).

### Absorption Spectrophotometry

A Shimadzu UV-3101PC spectrophotometer was used. Spectra were recorded at between 200 and 700 nm by installing the slide directly onto the sample holder with barium sulfate (BaSO_4_), and using an APTMS-derivatized slide as a blank. The results of the diffuse reflectance were transformed to absorbance units using Kubelka-Munk’s conversion.

### Mass Spectrometry Analysis of the Water Drop After Irradiation

Mass spectra were generated using a MALDI-TOF Microflex (Bruker Daltonics Inc., MA, USA) instrument in a positive ion mode, using detection by reflection to obtain the spectra. Samples were mixed with a 2,5-dihydroxybenzoic acid (DHB) matrix in a 1:1 ratio, and 2 μL of each mix was deposited on a micro scout simple-holder slide.

Analysis of mass spectra was performed using the program Flex Analysis v. 2.2 version 2.2 (Bruker Daltonik GmbH, Germany).

## Results and Discussion

### Assembly Strategy

**Fig. 2:**
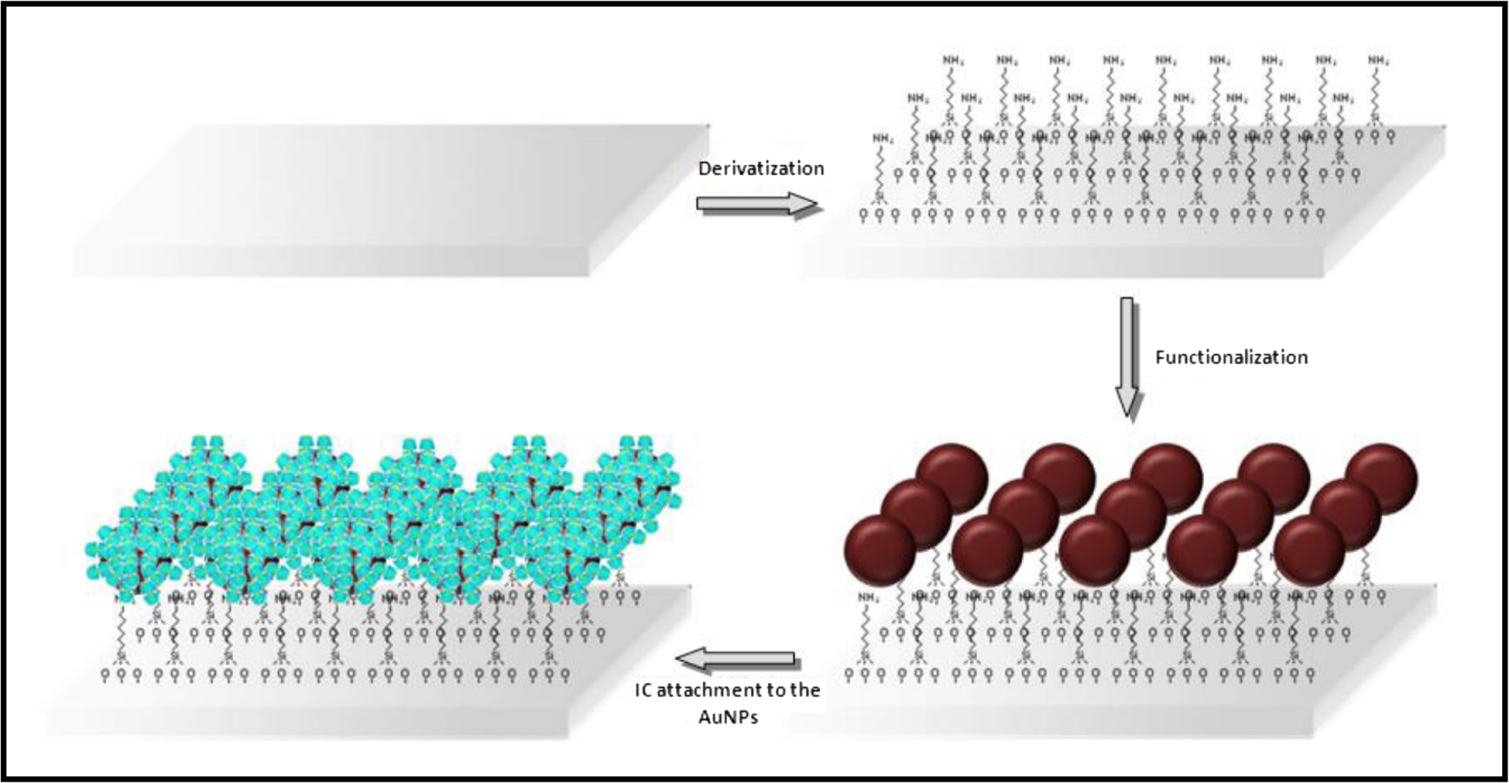
A schematic representation of functionalization steps of the glass. In *blue light* are represented the αCD-IC and in *brown* the AuNPs

A schematic representation of the functionalization stages of the glass is shown in Fig. 2. At first, the glass was activated using a piranha solution, hydrolyzing silane groups onto its surface and leaving –OH groups exposed. The surface was then derivatized with the APTMS organosilane, which binds to the hydroxyl groups via chemisorption with the methoxysilane groups. The glasses were then functionalized with AuNPs obtained following the Turkevich method [26] (characterization of the AuNPs colloidal is shown in the Additional file 1: Figure S2). The AuNPs were stabilized by the amine groups. Upon dipping of the silane-coated glass in the colloidal gold solutions, the AuNPs were immobilized on the surface. Due to the high affinity of gold to amine groups of the silane exposed on the outer surface of the glass, AuNPs were bound to the surface [31]. Finally, the inclusion compound, α-CD with octylamine (dissolved in DMSO), was deposited onto the functionalized glasses containing the AuNPs.

### AFM Monitoring the Effect of Irradiation on ICs Supported on Functionalized Glass Slides

**Fig. 3:**
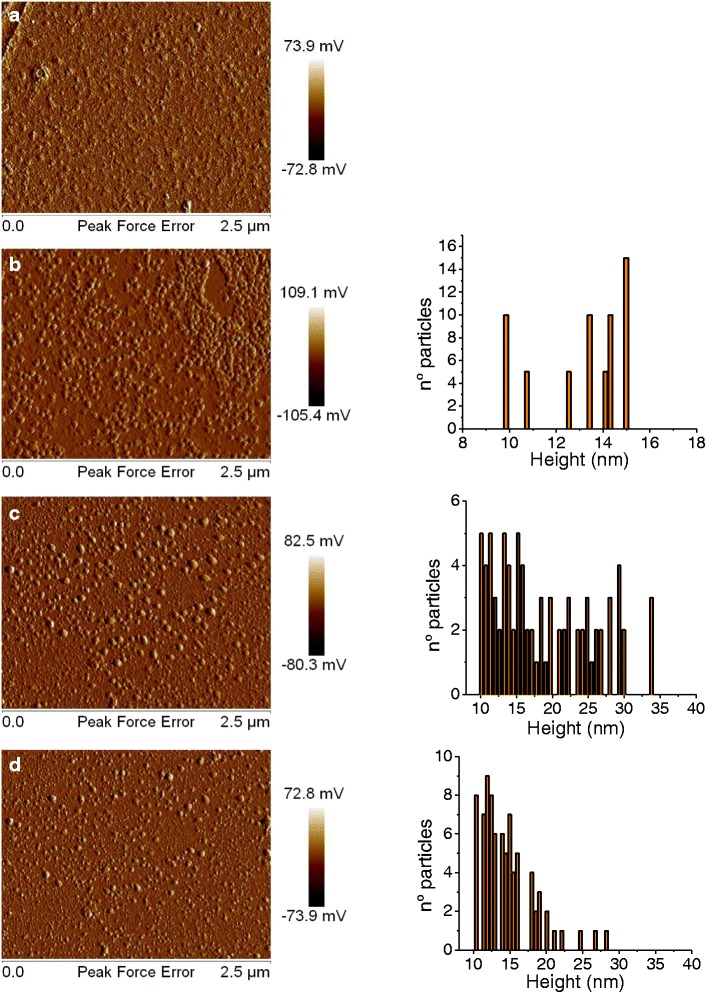
AFM micrographs of peak force error and a height histogram during each stage of functionalization. **a** Derivatized slides (glass/Si). **b** Slides functionalized with AuNPs (glass/Si/AuNPs). **c** Glass functionalized with AuNPs covered by IC with added water prior to irradiation (glass/Si/AuNPs/IC). **d** Glass/Si/AuNPs/IC after 5 h of irradiation

Figure 3 shows AFM micrographs of each stage of the glass functionalization. Figure 3a shows a micrograph of the derivatized glass, a rough surface. Figure 3b shows the AuNPs-functionalized glass, comprising primarily spherical particles that are deposited homogeneously on the derivatized glass. The histogram, calculated for a population of 60 particles, shows the height range of the AuNPs to be between 10 and 15 nm. Figure 3c shows the functionalized glass with AuNPs conjugated to ICs. An increase in the diameter and height of the AuNPs was observed when compared with the previous stage. The histogram shows a population of particles showing a preservation of heights of between 10 and 15 nm, denoting AuNPs not covered with IC, and a new population of particles with heights of between 15 and 34 nm. These higher particles denote AuNPs covered by IC.

To study the effects of the irradiation on the functionalized glass slides containing the IC-covered AuNPs, a drop of water was added to the sample, which was then irradiated continuously and monitored by recording AFM images during the 5 h of the irradiation process. The micrograph (Fig. 3d) shows that after irradiation, a drastic decrease in the height of the AuNPs covered by IC occurred (the histogram shows absence of structures in the range of 22 to 34 nm) which indicates that IC has been detached from the surface. However, the particles remained anchored and not aggregated on the surface after irradiation.

Additionally, we observed an increase in the population of particles at between 10 and 15 nm, which are similar to the heights obtained in the glass functionalized with AuNPs only. This finding is consistent with the theory that the majority of nanoparticles lost all of the parts of overlaid IC due to the plasmonic heat generated by the irradiation.

This phenomenon was detected within the first 15 min of the experiment (time of registering an AFM micrograph). Micrographs of functionalized glass with AuNPs covered with IC in the presence of water but with no irradiation at 0 h and at 5 h were taken as controls. There was no evidence of any difference in the micrographs or histograms between these time points. These results confirm that the variation in height of the particles was due only to the effect of plasmonic heating and rule out a possible degradation over time due to dissolution (Additional file 1: Figure S3 and S4).

In order to determine whether the laser increase the temperature in the experimental device, we determined this parameter in the water drop on the functionalized glass surface (with or without AuNPs) observing increments of about 4 °C after irradiation (Additional file 1: Figure S5). Notably, the local temperature around the AuNPs should reach 117 °C, which is consistent with thermal studies on solid IC that show reversible phase changes attributed to the movement of the guest molecule in CD cavities (Additional file 1: Figure S6). This finding is consistent with studies showing that it is possible to reach temperatures >100 °C via photothermal processes in liquid conditions [32].

### Absorption Spectrophotometry

To complement the AFM analysis, each stage of the glass functionalization was characterized by diffuse reflectance spectrophotometry.

**Fig. 4:**
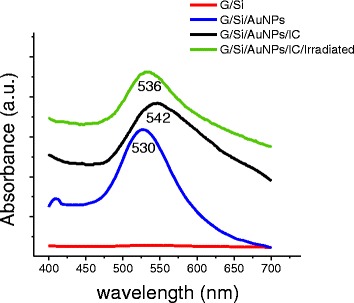
Absorption spectra of the glass during each stage of functionalization and at 5 h (after irradiation)

Figure 4 shows the absorbance spectra of each stage of glass functionalization. The silane-derivatized glass shows a baseline without absorbance. The spectrum of the glass functionalized with AuNPs shows a plasmonic band at 530 nm. The plasmon bathochromic shift to 520 nm (characteristic for colloidal particles stabilized with citrate molecules) from 530 nm may be explained as an effect of the change of the dielectric medium or the environment of the nanoparticles.

In the absorption spectrum of the glass functionalized with AuNPs covered with IC,a bathochromic shift was again observed from the maximum of 530 to 542 nm, which is generated by a change in the immediate environment of the AuNPs conjugated to IC. Additionally, an asymmetrical broadening of the plasmonic band was observed, which is indicative of particles with a large size polydispersity due to an uneven deposition of IC over the surface of the AuNPs.

Finally, absorption spectrum of the irradiated sample was taken and showed a hypsochromic shift from 542 to 536 nm, which may be attributed to a partial release of IC from the surface of the AuNPs.

AFM and UV-Vis analyses demonstrated unequivocally the release of IC from the surface of the AuNPs when irradiated with a green laser.

### Mass Spectrometry Analysis of the Water Drop After Irradiation

To elucidate whether the irradiation caused the disintegration of the IC, a mass spectrometry MALDI-TOF analysis was performed using a sample that contained a collection of drops obtained from the surface of functionalized glass slides previously irradiated for 20 min.

**Fig. 5:**
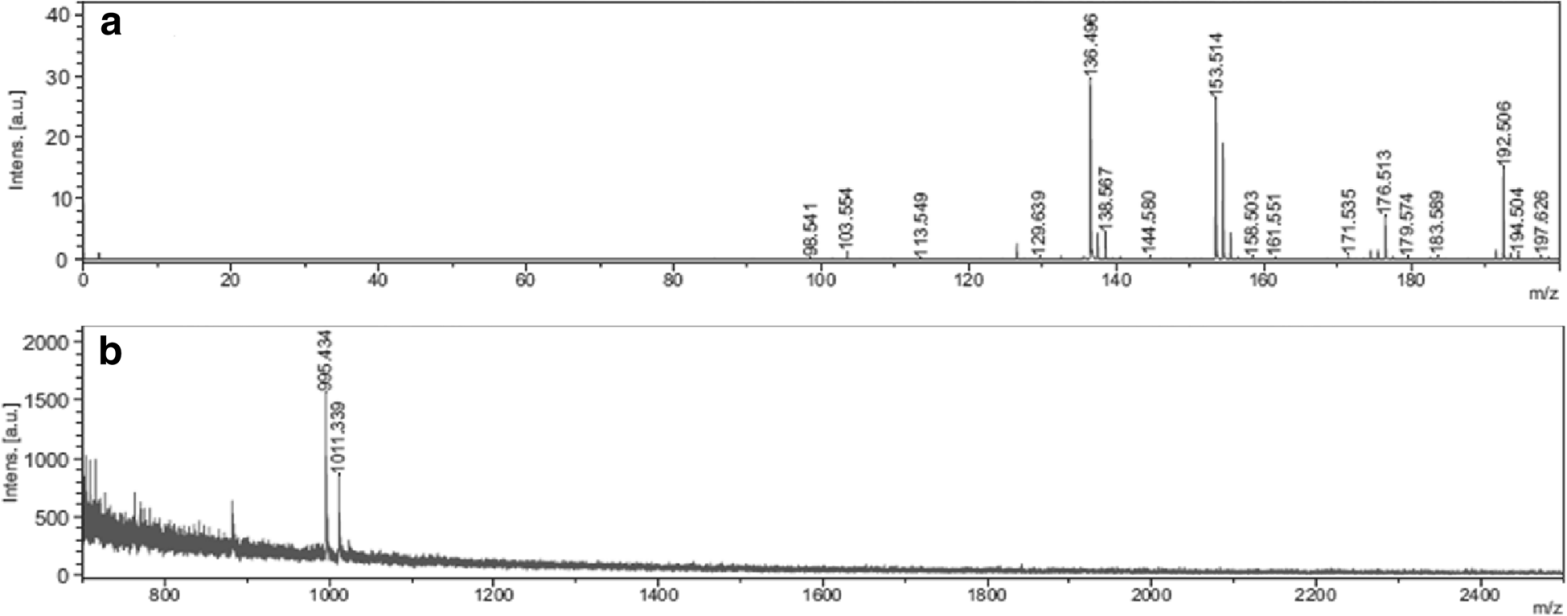
Mass spectra of a collection of drops of a parallel irradiation experiment in matrix of 2,5-dihydroxybenzoic acid (DHB) at interval **a** m/z 0–200 and **b** m/z 700–2500

Figure 5a shows the mass spectra of a collection of drops of a parallel irradiation experiment. In the interval m/z 0–200, a small signal at m/z 129 is observed that is indicative of residual OA (C_8_H_19_N; 129 g/mol). In the interval m/z 700–2500 in Fig. 5b, only the presence of α-CD (C_36_H_60_O_30_; 972 g/mol) is observed, with signals at m/z 995 and m/z 1011, which may correspond to the sodium and potassium species [M + Na]^+^ and [M + K]^+^, respectively. The presence of cyclodextrin and the absence of OA in the aqueous phase corroborate the disintegration of ICs. This finding is consistent with the absence of the corresponding signal of dimmer IC (nominal mass of 2073 g/mol) or fragmented IC (nominal mass of 1101 g/mol; Additional file 1: Figure S7).

**Fig. 6:**
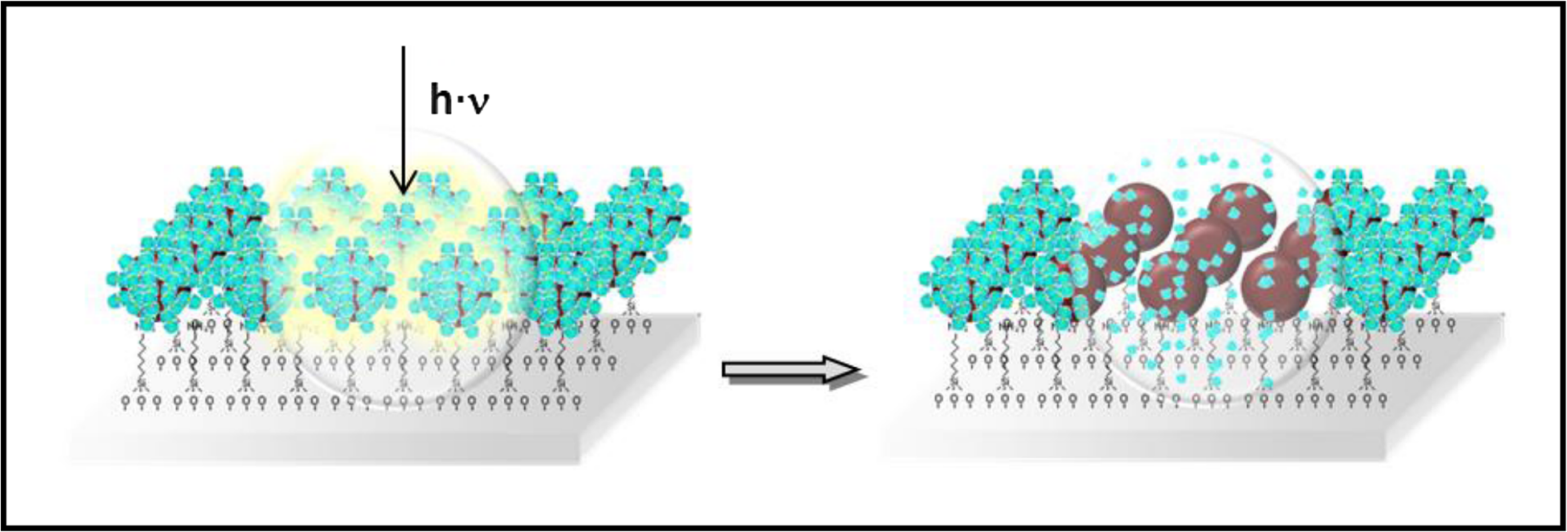
A schematic representation of the process of separation of ICs via irradiation. In *blue light* are represented α-CD-IC and in *brown* the AuNPs

Figure 6 summarizes the results of the irradiation process of the AuNPs-functionalized glass covered with IC and a water drop, obtained via AFM, absorption spectrophotometry, and MALDI-TOF analyses. Irradiation of the glass causes the disintegration of ICs. Cyclodextrin is dissolved in the aqueous phase, and octylamine remains anchored to the surface of AuNPs.

## Conclusions

In this study, the functionalization of AuNPs glass slides was possible, and their overlay with IC was successful and was evaluated by AFM and UV-Vis spectrophotometry during every step of the glass assembly (activation, derivatization, functionalization, and coating of AuNPs with IC).

It was possible to confirm the release of ICs from the AuNPs surface, due to the plasmonic heat of the irradiated nanoparticles, by observing a decrease in the height of the AuNPs using AFM and by observing one hypsochromic shift of the plasmon band using UV-Vis spectrophotometry.

Analysis of the water drop on the surface of the irradiated glass using MALDI-TOF verified that the plasmonic heat led to the loss of the guest because the nominal mass corresponded only to α-CD and traces of OA.

This study provides the first evidence of the disintegration of an inclusion compound due to local heating of AuNPs by laser irradiation at a wavelength tunable with the plasmon, thus separating ICs from the AuNPs surface. By using other methodologies as NMR is difficult to set up the irradiation and the observation at the same time which could lead to the reversion of the process forming the IC and or the AuNP-IC system. The release of the guest molecule may be relevant in the field of drug delivery where spatial and temporal control of the release of a guest from an inclusion complex is desired. Moreover, we designed a methodology that could serve as a screening method to test the release of drugs based on the photothermal effect.

## Additional file

Additional file 1: Characterization of citrate-stabilized colloidal AuNPs. AFM Micrographs of glass/Si/AuNPs/IC at different times of laser irradiation. Thermogram IC. Control details. Control 1. AFM Micrographs and height histograms of functionalized glass with AuNPs covered in IC, with addition of water drop at time zero and after 5 h. Control 2. MALDI-TOF analysis of IC. Heating curve of the sample under laser irradiation. (DOCX 979 kb)
