# Supplementary material for: In Situ Visualization of the Local Photothermal Effect Produced on α-Cyclodextrin Inclusion Compound Associated with Gold Nanoparticles
Source: Nanoscale Res Lett. 2016 Apr 7;11:180. doi: 10.1186/s11671-016-1322-z (PMC4823228; doi:10.1186/s11671-016-1322-z)
Supplement: Additional file 1: — Characterization of citrate-stabilized colloidal AuNPs. AFM Micrographs of glass/Si/AuNPs/IC at different times of laser irradiation. Thermogram IC. Control details. Control 1. AFM Micrographs and height histograms of functionalized glass with AuNPs covered in IC, with addition of water drop at time zero and after 5 h. Control 2. MALDI-TOF analysis of IC. Heating curve of the sample under laser irradiation. (DOCX 979 kb) [file 11671_2016_1322_MOESM1_ESM.docx]

**Electronic Supplementary** Material

**IN SITU VISUALIZATION OF THE LOCAL PHOTOTHERMAL EFFECT PRODUCED ON**

**α-CYCLODEXTRIN INCLUSION COMPOUND ASSOCIATED WITH GOLD NANOPARTICLES**

Nataly Silva^1^, Camila Muñoz^1^, Jordi Díaz-Marcos^2^, Josep Samitier^3^, Nicolás Yutronic^1^, Marcelo J. Kogan^4^*, Paul Jara^1^*

^1^*Department of Chemistry Universidad de Chile, Las Palmeras 3425, Santiago, Chile.*

*^2^Centro científico y tecnológico (CCiT) Universidad de Barcelona, Lluís Solé i Sabaris 1-3, Barcelona 08028, Spain.*

^3^*Nanobioengineering Laboratory, Institute for Bioengineering of Catalonia (IBEC), BaldiriReixac,10-12, Barcelona, 08028, Spain*. *Centro de Investigación Biomédica en Red de Bioingeniería, Biomateriales y Nanomedicina (CIBER-BBN),Maria de Luna, 11, 50018, Zaragoza, Spain.Department of Electronics, Barcelona University (UB), Martí I Franques, 1, Barcelona, 08028,Spain*

^4^*Department of Pharmacological and Toxicological Chemistry, Universidad de Chile, Sergio Livingston 1007, Santiago, Chile* and Advanced Center for Chronic Diseases (ACCDiS).

*Corresponding authors*: pjara@uchile.cl; mkogan@ciq.uchile.cl*

**
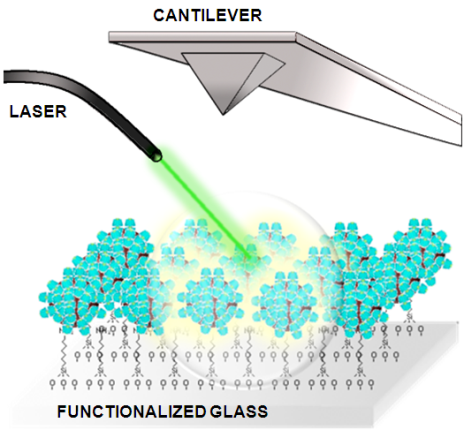
**

**Figure S1.** Schematic representation of monitoring by AFM of the functionalized glass irradiated with green laser.

**a)**





**b)**

**Figure S2.** Characterization of citrate- stabilized colloidal AuNPs. A) TEM micrograph (left) and Histograms (right), b) UV-visible absorptionspectra.

Figure 2a presents a TEM micrograph and the corresponding size-distribution histogram of citrate-stabilized colloidal AuNPs. A homogeneous distribution in size and shape is observed. The histogram, which was obtained from a population of 100 particles, indicates an average diameter of 12 ± 1.3 nm.

Figure 2b presents the absorption spectra of AuNPs. An absorption maximum is observed at 520 nm, corresponding to the band that is characteristic of plasmon resonance for spherical gold nanoparticles near to 10 nm in diameter.


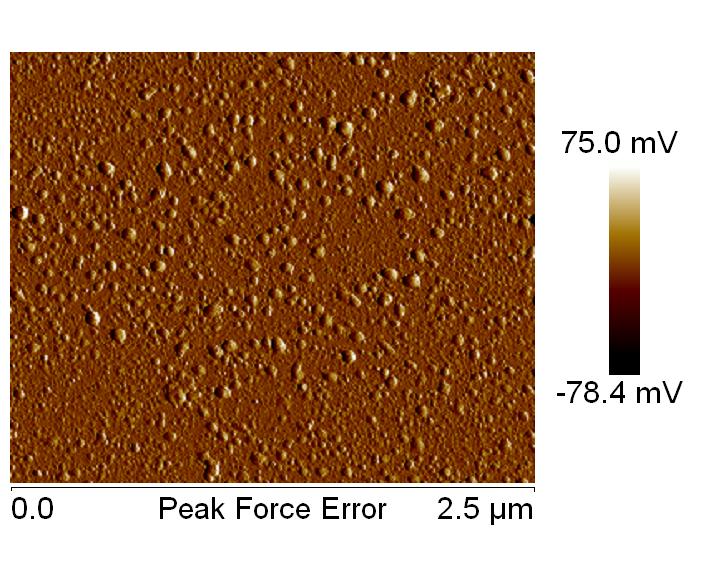


**15 min**


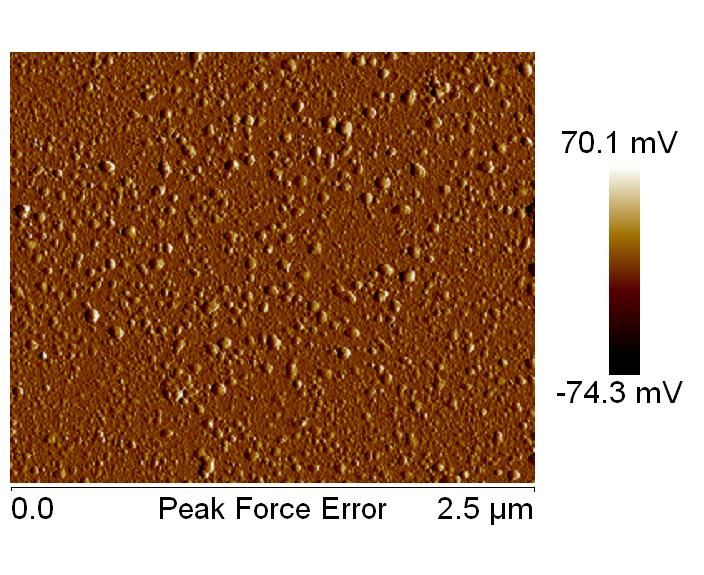


**2 hrs**


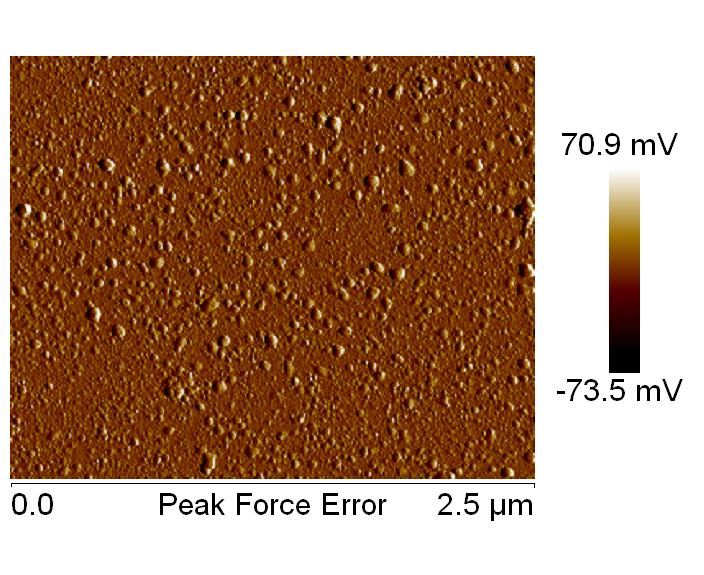


**4 hrs**

**Figure S3.** AFM Micrographs of peak force error and of heights histograms of glass/Si/AuNPs/IC at different times of laser irradiation.

**Control 1. Unirradiated sample**

*
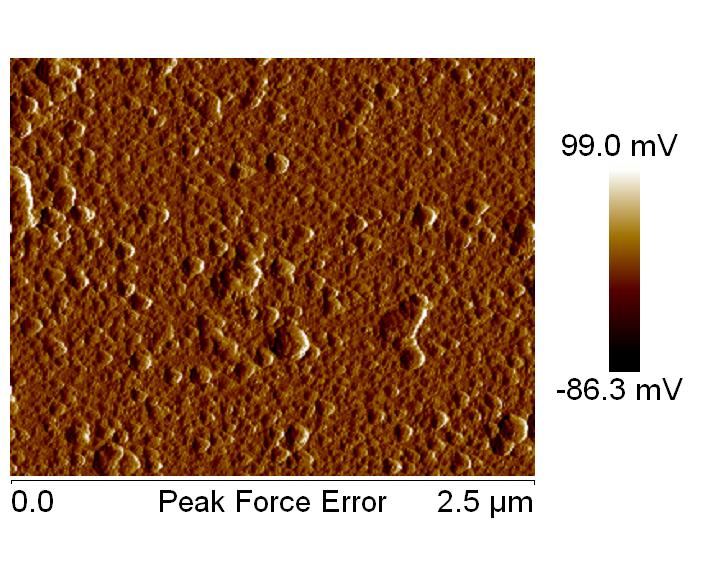
*

**a)**


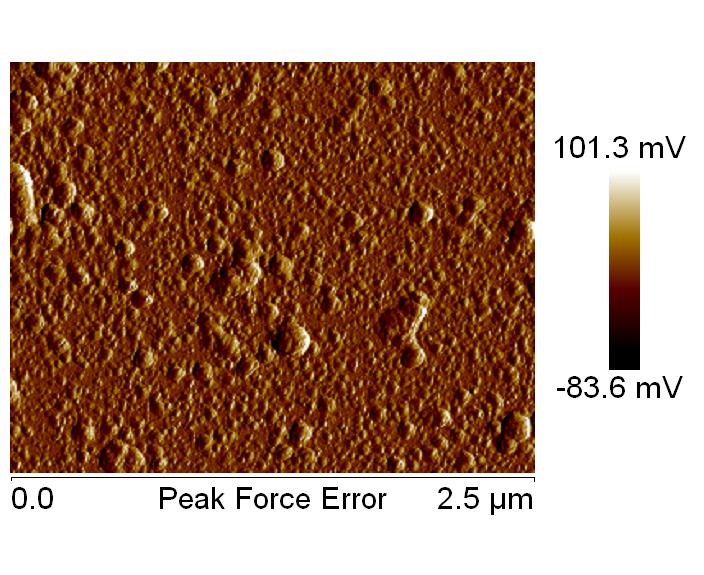


**b)**

**Figure S4.** AFM Micrographs of peak force error and height histograms of functionalized glass with AuNPs covered with CI, with addition of water drop. a) at zero time and b) after 5 hrs.

**Figure S5.** Heating curve of the sample under laser irradiation. The experiment has been performed at room temperature (20 °C).

**Figure S6.** Thermogram (DSC/TGA) of the α-cyclodextrin/octylamine inclusion compound.

Figure S6 shows the thermogram (Differential Scanning Calorimetry (DSC)/ Thermogravimetric Analysis (TGA)) of the IC sample. Two endothermic peaks near 114 and 124º C and two exothermic peaks at 119 and 130º C in DSC, are observed. TGA analysis shows absence of mass loss associated with these thermal events. The mass loss of 5.36% between 25 and 113° C is observed and attributed to the loss of water molecules from moisture of the IC.

The endothermic and exothermic peaks are attributed to conformational reversible changes of the host-guest system, probably due to migration movements of the guest molecule into the cyclodextrin cavity maintaining entire the supramolecular structure.

[1] Barrientos, L.; Lang, E.; Zapata-Torres, G.; Celis-Barros, C.; Orellana, C.; Jara, P.; Yutronic, N. *J.* Structural elucidation of supramolecular alpha-cyclodextrin dimer/aliphatic monofunctional molecules complexes. *Mol. Model.* **2013**, 19, 2119–2126.

**Control 2 Analysis of collection of water drops obtained from surface of functionalized glass with AuNPs covered with IC irradiated during 20 minutes.**

**a)**


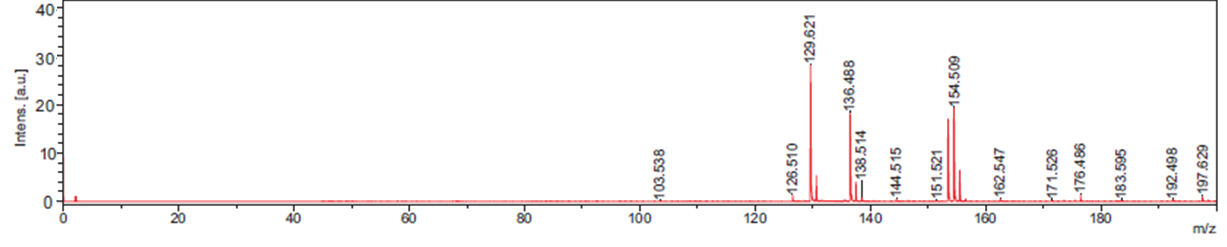


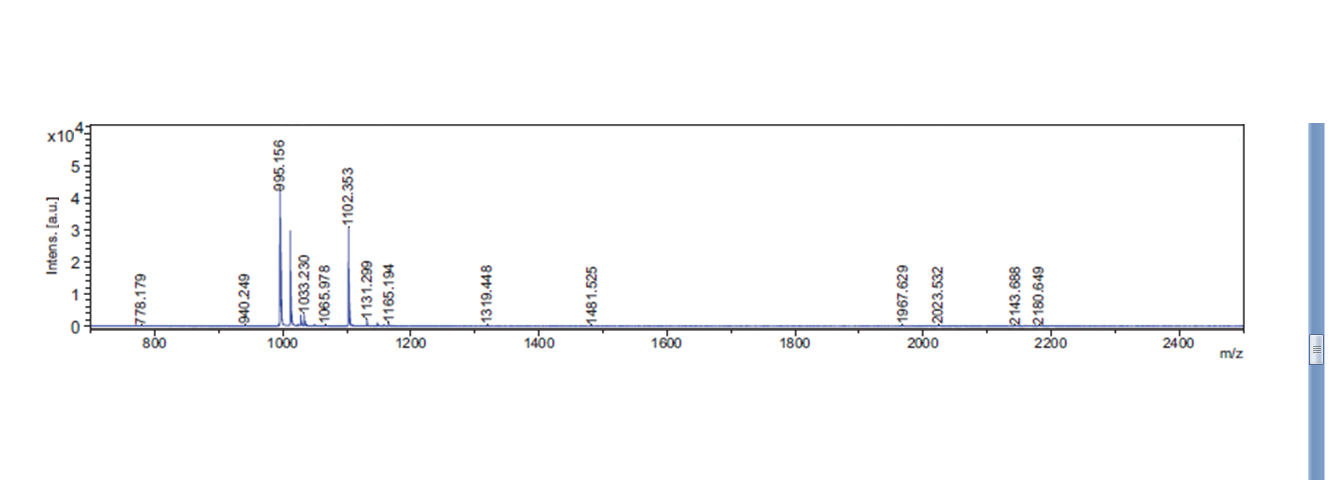


**b)**

**Figure S7.** Mass spectra of IC in intervals: a) m/z 0-200 and b) m/z 700-2500 (matrix DHB).

For the sample containing the IC constituted by two molecules of α-CD (C_36_H_60_O_30_; nominal mass 972 g/mol) and a molecule of octylamine (OA, C_8_H_19_N; nominal mass 129 g/mol), the presence of signals m/z 973 for α-CD, m/z 130 for OA and m/z 2074 for the species 2α-CD+OA (C_80_H_139_O_60_N; nominal mass 2073 g/mol), were observed. For the spectra acquired in the presence of DHB (Figure 5), the presence of signals attributed to α-CD and OA were observed, however, the presence of 2α-CD+OA (absence of the protonated form or adducts) was not observed. A signal m/z 1102 was observed, which may correspond to the presence of α-CD+OA (C_44_H_79_O_30_N; nominal mass 1101 g/mol).

The difference in the obtained stoichiometry through the MALDI-TOF analysis, in contrast with that obtained by ^1^H-RMN^1^ which gives a stoichiometry of host-guest 2:1 (2α-CD+OA) [1], may be explained by the fragmentation of the IC 2:1, generated by the asymmetry of the inclusion. As the OA molecule has a short alkyl chain, it is included asymmetrically in the CD cavity. A soft ionization then promotes the release of just a single cyclodextrin molecule.
